# Epigenetic Modulators of Monocytic Function: Implication for Steady State and Disease in the CNS

**DOI:** 10.3389/fimmu.2015.00661

**Published:** 2016-01-15

**Authors:** F. Nina Papavasiliou, Young Cheul Chung, Khatuna Gagnidze, Kaitlyn H. Hajdarovic, Dan C. Cole, Karen Bulloch

**Affiliations:** ^1^Laboratory of Lymphocyte Biology, The Rockefeller University, New York, NY, USA; ^2^Neuroimmunology and Inflammation Program, The Rockefeller University, New York, NY, USA; ^3^Harold and Margaret Milliken Hatch Laboratory of Neuroendocrinology, The Rockefeller University, New York, NY, USA

**Keywords:** monocytic, epigenetic, DNA methylation, histone modification, RNA editing

## Abstract

Epigenetic alterations are necessary for the establishment of functional and phenotypic diversity in the populations of immune cells of the monocytic lineage. The epigenetic status of individual genes at different time points defines their transcriptional responses throughout development and in response to environmental stimuli. Epigenetic states are defined at the level of DNA modifications, chromatin modifications, as well as at the level of RNA base changes through RNA editing. Drawing from lessons regarding the epigenome and epitranscriptome of cells of the monocytic lineage in the periphery, and from recently published RNAseq data deriving from brain-resident monocytes, we discuss the impact of modulation of these epigenetic states and how they affect processes important for the development of a healthy brain, as well as mechanisms of neurodegenerative disease and aging. An understanding of the varied brain responses and pathologies in light of these novel gene regulatory systems in monocytes will lead to important new insights in the understanding of the aging process and the treatment and diagnosis of neurodegenerative disease.

## Introduction

The mononuclear phagocyte system is a branch of the leukocyte family comprising macrophages, dendritic cells (DC), tissue macrophages, and microglia and their multiple subsets. While their diverse tissue and immune-specific functions have been the subject of much discussion and debate, the developmental origin of these cells remains largely undetermined (1).

**Figure 1:**
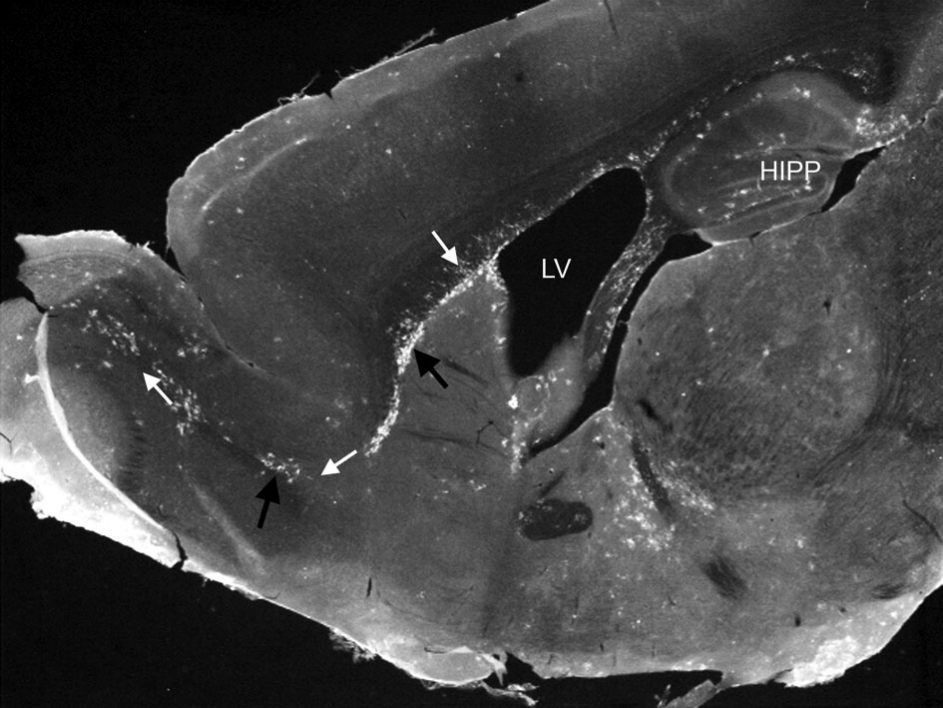
**Photomicrograph showing EYFP/CD11c^+^ cells (black arrows) in discreet regions of the adult steady-state CNS**. EYFP/CD11c^+^ cells are evident along the rostral migratory stream (white arrows), lining the subventricular zone of the lateral ventricles, the subgranule cell layer of the hippocampus, and within the circumventricular organs. The photomicrograph is illustrative of the data published by Bulloch et al. (5).

Cells that give rise to the mononuclear phagocyte system are embryonically derived from “blood islands” during embryonic development (2, 3). After development, common myeloid precursors derive from hematopoietic cells within the bone marrow, and egress into the bloodstream to migrate to the site of infections, where they differentiate into effector immune cells such as macrophages and DC. The origin of resident tissue macrophages and brain-resident monocytic cells is still controversial. Within the steady-state CNS, the origin, function, and turnover of subsets of monocytic cells, often collectively referred to as “microglia,” is a constantly evolving area of study, particularly due to issues defining this heterogeneous group as a single population. Until recently, it was thought that these were the only cell population, which performed immune surveillance in the brain, given that surveillance by peripheral monocytic leukocytes was considered limited at best. However, a recent study showed the presence of lymphatic vessels within the CNS demonstrating clear routes for leukocyte trafficking in and out of the brain (4). Additionally, studies of transgenic mice that express fluorescent protein markers for monocytic cells, in particular DC, clearly showed the presence of these leukocytes traversing discrete regions of the steady-state brain (Figure 1) (5). In multiple studies examining various immune challenges, results show that this EYFP^+^ cell population is comprising both peripherally and centrally derived monocytic cells with diverse functions (6–10).

Taken together, these findings demonstrate the CNS is subject to far greater immune surveillance than previously believed. This concept opens up new possibilities to define the function of monocytic cells in the brain and understand the vastly complicated relationship between cells of monocytic lineage and underlying etiologies of CNS neuropathology. The field of epigenetic regulation of immune function is one such area that begs further exploration, especially within the brain. The following will introduce this area and present what is currently known about this regulation in the context of CNS/immune function.

## DNA Methylation and Demethylation

DNA methylation is an epigenetic modification of genes that regulates genomic imprinting, X chromosome inactivation, cell fates during development (11), chromosomal stability, repression of transposable elements, and gene expression during the lifetime of the organism (12, 13). The majority of DNA methylation in mammalian genomes takes place within a CpG dinucleotide context (generating 5-methyl-CpG, or meCpG). However, meCpH methylation (where H = dA, dC, or dT) is also known to occur, notably in the adult mammalian brain (14).

Generally, the quantity of CpG methylation is inversely correlated with gene expression. CpG methylation can control transcription either locally [e.g., by occluding transcription factor binding sites within promoter regions (15) or by binding DNA within gene bodies and interfering with transcriptional elongation (14)] or globally (where methylation of CpG rich regions can lead to the recruitment of proteins that alter the chromatin state and result in general locus silencing). However, overall rules by which mCpG (or indeed hmCpG or mCpH) control transcription are not clear and attempts at generalizing specific findings are often controversial.

Recently, it has been convincingly demonstrated that DNA methylation can be reversed. Direct demethylation is not energetically favorable; thus, demethylation occurs in a number of steps, either through the direct deamination of meCpG to TpG and removal of the T:G mismatch by thymine DNA glycosylase (TDG)/Gadd45 (16–18) or through the enzymatic oxidation of meCpG to hmCpG (catalyzed by the TET family of proteins) and removal of the hydroxymethyl moiety through catabolism (formylation and carboxylation) and excision through DNA repair that again involves TDG (19).

Demethylation is crucial during development, to “erase” any paternal or maternal marks in the zygote, which are then “reset” by specific methyltransferases during imprinting, and remain grossly stable thereafter, in the vast majority of tissues. However, tissue-specific differentiation is associated with significant, if subtle, changes in the DNA methylome, and sets of “differentially methylated regions” or DMRs are known to be associated with plasticity and developmental changes. Some of these changes are rather dramatic: demethylation of a single open reading frame within a large imprinted region in astrocyte niches (leading to temporally restricted, bi-allelic expression of the Dlk1 gene) is essential for neurogenesis (20). Conversely, disturbances in imprinting implicate Dlk1 as a novel risk gene for experimental autoimmune encephalomyelitis (EAE) in mice (21). Most changes, however, are far less robust and result in levels of heterogeneity in patterns of DNA methylation (both hypo and hyper methylation of DMRs) that are much harder to link to clear mechanistic consequences.

Limited information is available about the DNA methylome in monocytes, or its plasticity during developmental transitions, such as differentiation from multipotent progenitors (MPP), lineage commitment, and aging (22, 23). Study by Ji and colleagues used comprehensive high-throughput array-based relative methylation (CHARM) analysis and revealed striking epigenetic plasticity, resulting in increased overall methylation upon lymphoid relative to myeloid commitment. Most DMRs distinguishing MPP cells from common lymphoid progenitors (CLP) lost methylation during this step of early lymphoid commitment. In contrast, in common myeloid progenitors (CMP) in the earliest step of myeloid commitment displayed substantially more hypermethylated than hypomethylated DMRs. Interestingly, at the next step of lineage commitment, both of these trends were reversed and 15-fold more DMRs showed gain of methylation upon the transition from CLP to DN1, and nearly all DMRs showed loss of methylation on transition from CMP to GMP. Comparing DN1 to GMP, two populations similarly differentiated toward lymphoid and myeloid fates, respectively, there were eightfold more DMRs with higher-level methylation in DN1 cells, suggesting a skewing toward greater methylation in lymphoid compared to myeloid hematopoiesis. These observations were in concert with data obtained from Dnmt1-hypomorphic mice (which are unable to maintain CpG methylation properly) showing normal myeloid, but diminished lymphoid development (24). Moreover, inhibiting DNA methylation in *in vitro* system using 5-aza-29-deoxycytidine promotes myeloid versus lymphoid specification, providing a mechanism for the myeloid skewing observed in Dnmt1 hypomorphs.

Nothing is known about the status of methylation in cells of the monocytic lineage in the brain (bDCs or microglia). Generally, what is known comes from a handful of studies of monocyte to DC differentiation *in vitro* (25) or human monocyte to macrophage differentiation *in vivo*, in the context of pregnancy (26). *In vitro*, regions around specific demethylated CpGs could be associated with alterations in transcription factor binding sites for factors important to maturation (e.g., Jun, Bcl11a in the monocyte to DC transition), resulting in robust changes in methylation of a handful of promoters (SRC, PLEKHG6, and ITGB2), which were in turn correlated with increased transcription. *In vivo*, similar pattern changes are also visible in the monocyte to macrophage comparison, where the small magnitude of changes (e.g., 25% decrease in methylation in the Lag3 promoter, or 50% increase in the Ilib promoter) results in substantial transcriptional changes, through mechanisms that are not immediately obvious.

However, mild or mechanistically unclear, methylation changes in DNA in macrophages have been associated with inflammation [e.g., pathogen infection (27) or environmental changes – e.g., smoking (28)], memory to infection [e.g., endotoxin tolerance (29)], and aging (23). Though currently methylome maps are not available for cells of the monocytic lineage in the brain, it is expected that these will be different within different cell subsets or types (i.e., microglia, macrophage, and bDC), and will produce distinct patterns under different conditions. Conversely, mild differences in methylation or demethylation mediators [e.g., deaminases such as AID or APOBEC2 – (16), or hydroxylases such as the TET family of proteins] might result in patterns “at risk” for disease, or alter disease onset and manifestations (30).

## Histone Modification

While cytokines and chemokines are believed to be initially responsible for inducing monocyte development in the adults, every phase of monocytic differentiation is ultimately regulated by a specific set of transcription factors, which confer proper expression of lineage-specific genes at the appropriate time during development and differentiation or in response to immune challenge. Histone modifications and chromatin remodeling are evolutionarily conserved regulatory mechanisms that can repress of facilitate gene expression in response to environmental and developmental stimuli (31). Post-translational modifications of histone proteins, which are major constituents of chromatin, include acetylation, methylation, phosphorylation, ubiquitination, ribosylation, and sumolation. Such modifications have been shown to affect chromatin structure and make regulatory sequences more or less accessible for transcription factor and co-factor binding. As a result, covalent modifications of histone proteins have a major impact on the gene expression (31). Another important characteristic of histone modifications is their dynamic nature and responsiveness to environmental changes, which places them at the interface of genes and environment. Thus, it is not surprising that histone modifications have been shown to regulate the migration and differentiation of monocyte-derived cells in response to injury and infection (29).

Histone modifications have been shown to affect an important step in monocyte-mediated immune function, which is migration to the site of inflammation and to the lymph nodes. Signaling through chemokine receptors, such as CCR2, CCR5, and CCR7, and the expression of their ligands are critical for the proper immune response (32). For example, CCL2, also referred to as monocyte chemoattractant protein-1 (MCP-1), is involved in directing the egress of monocytes into the bloodstream and their migration to the sites of inflammation (33, 34). Excessive CCL2 secretion has been linked to many inflammatory diseases, whereas a lack of expression severely impairs immune responsiveness (35).

Under non-inflammatory conditions, the Ccl2 locus is transcribed at low level, but rapid induction of gene expression occurs on exposure of cells to various proinflammatory stimuli. NF-kB transcription factor has been shown to be the master regulator of LPS-induced activation of CCL2 and recent report demonstrate that the coactivator, IkBz, is also critical for the expression of this chemokine in the macrophages (36). Specifically, the direct recruitment of IkBz to the proximal promoter of Ccl2 and induction of H3K4 trimethylation seem to be required for the production of CCL2 in macrophages, since IkBz-deficient macrophages exhibited impaired secretion of CCL2 when challenged with LPS or peptidoglycan. Additionally, IkBz-deficient mice showed significantly impaired CCL2 secretion and monocyte infiltration in an experimental model of peritonitis (36).

An interesting mode of regulation has been described for another chemokine receptor gene, Ccr5. Although monocytes display high levels of CCR5, the expression is lost as cells start to differentiate into macrophages and DC. Such rapid downregulation of Ccr5 expression is believed to be achieved because of multivalent chromatin state characteristic of its promoter (37). Specifically, it has been shown that Ccr5 promoter is covered with high levels of activating histone mark, AcH3, as well as relative high levels of repressive modifications, H3K9me3 and H3K27me3. Moreover, the histone modifications in monocytes are accompanied by relatively high levels of DNA methylation of Ccr5 promoter. The fact that monocytes display high amounts of repressive marks in conjunction with histone acetylation may reflect the potential to rapidly shut down Ccr5 transcription upon differentiation.

Repressive histone modification, H3K27me3, has also been shown to accumulate at the promoter of Ccr7 gene in monocyte-derived DC but not in conventional DCs (38). As a result, moDCs express very low levels of CCR7 and do not migrated to lymph nodes as effectively as cDCs following inhaled allergen challenge. This example demonstrates how histone modification of the chemokine receptor can control the migration and therefore the function of monocyte-derived DCs *in vivo*.

After arriving at the site of infection, monocytic cells differentiate into effector cells, such as DC and macrophages. A well-orchestrated gene expression program is necessary to activate lineage-specific genes and at the same time repress transcripts characteristic of progenitors. A genome-wide mRNA expression profile and map of three major histone modifications (AcH3 and H3K4me3-permissive and H3K27me3-repressive mark) associated with gene expression in monocytes, macrophages, and DCs have demonstrated that specific sets of histone modifications are employed to execute such transcriptional programs (39). For example, during differentiation, the H3K4me3 levels decreased on monocyte-specific CD14, CCR2, and CX3CR1 but increased on DC-specific TM7SF4/DC-STAMP, TREM2, and CD209/DC-SIGN genes as well as genes associated with phagocytosis and antigen presentation. Moreover, in macrophages and DCs, H3K4me3 levels increased in a large genomic cluster of proinflammatory and chemotactic CC chemokines on chromosomes 17q11.2 and 16q13.

Another study has demonstrated the role of H3K4 methylation in the expression of specific proinflammatory cytokines in macrophages. Xia and colleagues (40) have shown that Ash1l, a H3K4 methyltransferase, suppressed interleukin-6 (IL-6), and tumor necrosis factor (TNF) production in toll-like receptor (TLR)-triggered macrophages, protecting mice from sepsis. Moreover, Ash1l-silenced mice were more susceptible to autoimmune disease as a result of enhanced IL-6 production. Further analysis revealed that Ash1l induced H3K4me modification at the Tnfaip3 promoter via its methyltransferase activity thus controlling innate IL-6 production and suppressing inflammatory autoimmune diseases, providing mechanistic insight into epigenetic modulation of immune responses and inflammation.

Histone modification plays the role not only in differentiation of macrophages but also in polarization of their phenotype into M1 and M2 subtypes. Whereas M1 macrophages are proinflammatory, M2 macrophages are associated with response to anti-inflammatory reactions and tissue remodeling. Deletion of histone deacetylase HDAC9 in an atherosclerosis mouse model (LDLr^−/−^, low density lipoprotein receptor knock out) reduced atherosclerosis and resulted in polarization of macrophages toward M2-like phenotype as well as upregulation of lipid homeostatic genes and downregulation of inflammatory genes (41). The upregulation of M2 and downregulation of M1 genes in HDAC9-deficient macrophages were hypothesized to be through PPAR-γ pathway since the expression of PPAR-γ in double KO macrophages was increased compared with single KO macrophages and quantitative ChIP assays demonstrated increased levels of total H3, H4, H3K9 at the promoter of PPAR-γ. These experiments support the concept that increased HDAC9 expression in macrophages may be atherogenic via suppression of cholesterol efflux and generation of alternatively activated M2 macrophages.

M2-macrophage activation is mediated by IL-4 and/or IL-13 and is associated with parasite infections and allergic inflammation. It has been shown that continuous IL-4 signaling leads to transcriptional activation of STAT6 and subsequent increase in the levels of demethylase Jmjd3 (42). As a result, increased Jmjd3 contributes to the decrease of inhibitory H3K27me2/3 marks at the promoter of M2 marker genes and promote M2 phenotype. The authors confirmed the decrease in H3K27me2/3 and increase in Jmjd3 recruitment to M2 marker genes by *in vivo* studies using a Schistosoma mansoni egg-challenged mouse model, a well-studied system known to support an M2 phenotype.

The same immune challenge was used to demonstrate the role of HDAC3 in M2 polarization (43). HDAC3 binds genome-wide and acts at a subset of IL-4 target gene enhancers to restrict deposition of activating histone marks. Macrophages lacking histone HDAC3 display a polarization phenotype similar to IL-4-induced alternative activation and are hyperresponsive to IL-4 stimulation. In addition, exposure to Schistosoma mansoni eggs of mice lacking HDAC3 prevented development of pulmonary inflammation. Interestingly, HDAC3-deficient macrophages display deficit in response to LPS and are unable to activate the expression of large number of inflammatory genes normally upregulated by LPS (44).

Lysine demethylase JMJD3 has also been shown to be involved in the inflammatory response. In macrophages, JMJD3 expression is rapidly induced by proinflammatory stimuli, and it is recruited to the transcription start sites (TSSs) of LPS-induced genes, where it participates directly in the transcriptional response (45, 46). To demonstrate whether this activation of transcription is achieved through the demethylation of H3K27me3 at target gene promoters, Kruidenier and colleagues developed and used selective JMJD3 inhibitors GSK-J4 and GSK-J5 to modulate LPS-induced immune response in human primary macrophages. Administration of GSK-J4 significantly reduced the expression of ~50% of LPS-driven cytokines as assessed by PCR array, including TNF-α. In addition, chromatin immunoprecipitation (ChIP) studies confirmed that GSK-J4, but not GSK-J5, prevented the LPS-induced loss of H3K27me3 associated with the TNF-α TSS and blocked the recruitment of RNA polymerase II to this locus (47).

Several studies have explored the therapeutic potential of HDAC inhibitors as anti-inflammatory agents. Both *in vivo* and *in vitro*, various HDAC inhibitors, suberoylanilide hydroxamic acid (SAHA), trichostatin A (TSA), and sodium valproate (VPA) have been shown to block the secretion of proinflammatory cytokines, such as TNF-α, IL-1, IL-6, and IL-12.

Moreover, HDAC inhibitor administration to mice was found to ameliorate the autoimmune manifestations of graft-versus-host disease, systemic lupus erythematosus, concanavalin A-induced hepatitis, EAE, rheumatoid arthritis, and colitis (48–51). On a mechanistic level, Nenconi and colleagues demonstrated that HDAC inhibitors, sodium VPA and MS-275, interfered with DC differentiation from monocytic cells (52). DCs exposed to HDAC inhibitors have reduced expression of CD1a, a DC hallmark, CD80 and CD40, costimulatory molecules, and CD83, which is typically expressed on mature DCs. Consistent with anti-inflammatory function of HDAC inhibitors, the treatment profoundly impaired the DC secretion of the proinflammatory cytokines TNF-α and IL-6, IL-12 as well as IL-10 production. Finally, DCs grown in the presence of VPA or MS-275 had impaired immunostimulatory capacity and migration to CCL19.

Recently, the concept of “trained immunity” was introduced, which postulates that innate immune responses launched against initial infection may afford protection against reinfection. The evidence for such “trained immunity” was provided by infecting mice lacking functional T and B lymphocytes (to rule out the contribution of adaptive immunity) with *Candida albicans* and demonstrating protection against reinfection in a monocyte-dependent manner (53). Monocyte training by fungal cell wall β-glucans was associated with stable changes in global histone trimethylation H3K4, but not of H3K27me3. Genome-wide RNA-seq analysis following β-glucan treatment confirmed a strong correlation between the increase in H3K4me3 occupancy and the increase in gene expression. More specifically, H3K4me3 was elevated at the promoters of important target genes such as the proinflammatory cytokines TNF-α, IL-6, and IL-18 after β-glucan treatment and subsequently, increased gene transcription of TNF-α and IL-6 mRNA upon restimulation was observed (53). These data clearly demonstrate the important role of stable and long-lasting histone modification in the “trained immunity.” Moreover, it has been demonstrated that “endotoxin tolerance,” a form of innate memory in which the initial stimulation of monocytes or macrophages with the TLR4 ligand LPS causes these cells to enter a long-term refractory state, also depends on H3K4me3 epigenetic mark (54). The restimulation of tolerant macrophages with LPS produces two different gene-expression profiles: one set of “tolerized” genes show diminished or abolished expression, whereas the expression of a second group of “non-tolerized” genes is increased or remains unchanged. Although the transcription-activating H3K4me3 and H4Ac marks are present on the promoters of both tolerized and non-tolerized genes, following reinfection they are only maintained on the promoters of the non-tolerized genes whereas the tolerized genes, including those encoding inflammatory cytokines, remain devoid of this mark (54).

Together, these examples demonstrate that the ability of monocytic cells to migrate to the site of infection and differentiate into effector cells in response to environmental cues greatly depends on their capacity to rapidly upregulate or shut down large sets of genes. Proper regulation of chromatin states through histone modifications is critical in executing these functions.

## RNA Dynamics

RNA metabolism is a complex process that encompasses RNA transcription, pre-mRNA processing/splicing, transport, location, stability, and/or translation of mature mRNAs and mRNA decay. Epigenetic regulatory mechanisms in RNA metabolism are known to orchestrate virtually all steps of the mRNA life cycle ranging from pre-mRNA splicing to mRNA degradation. Remarkably, these regulators can coordinate the expression pro-inflammatory and anti-inflammatory molecules to initiate, maintain, and resolve immune response in monocytic cells (55, 56). These epigenetic processes are controlled by numerous regulators, including microRNA (miRNA), long non-coding RNA (lncRNA), RNA-binding proteins (RBP), and RNA editing enzymes (57–59). These regulators can promote mRNA stabilization/degradation or prevent mRNA translation. In the following, we will discuss the role of miRNA, lncRNA, RBPs, and RNA editing enzymes as epigenetic regulators, and the role of RNA metabolism in cells of monocytic lineage.

### microRNA

microRNAs are potent new players in epigenetic regulation. miRNAs can respond quickly to environmental stimulation and act to regulate many different systems. For example, miR-27a has been shown to be involved in changes in monocyte differentiation following incidents of high alcohol consumption (60). In this study, healthy volunteers showed significant changes in the populations of circulating monocytes and these changes were replicated *in vitro* in the presence of alcohol. Alcohol-mediated miR-27a upregulation led to polarization and activation of circulating monocytes.

The vast variety of different miRNAs can have diverse effects upon cells of monocytic lineage. miR-124 can suppress the immune response in microglia by modulating P65 activation. Interestingly (61), miR-124 is also responsive to the environment: its strongest phenotype is the result of long-term opioid treatment. The NF-kB pathway can be silenced by the expression of miR-203 in microglia (62). Suppression of this pathway can protect against microglial death and degeneration after brain injury and could potentially be a target of therapies aimed at reducing the effects of neurodegenerative diseases.

The modulation of macrophage phenotype is important for their proper function; dysregulation of their phenotype can contribute or even cause pathological conditions and damage. The role of miRNA in the regulation of the switch from the M1 inflammatory and M2 repair phenotypes in macrophages is only recently coming to light. miR-21 has been shown to be an important player in the pathway that causes this switch. It acts to regulate the proinflammatory response and can inhibit the NF-kB pathway through interaction with the colony-stimulating factor (CSF) pathway (63). miRNAs also play a role in the recruitment of macrophages themselves. miR-26a also acts upon the CSF and prevents the recruitment of macrophages in a hepatocellular carcinoma cell line (64).

There is increasing evidence that miRNAs play a key role in the progression of Alzheimer’s disease (AD). Further exploration into the role of miRNAs in AD could lead to novel models and eventual treatments. One of the most promising miRNAs involved in AD is miR-155 (65). It has been shown to be a key regulator of T lymphocyte function during inflammation. Mice lacking miR-155 are shown to have reduced T cell-mediated immune responses, and T cells from these animals are more likely to be Th2 cells that act to reduce the proinflammatory response. T cell infiltration of the brain is one of the key dysfunctions in the progression of AD. In the 3xtg triple transgenic AD model mouse, upregulation of miR-155 has been linked with an increase in the severity of the AD phenotype (66). This event appeared at the very beginning of the onset of AD, before the appearance of plaques and concurrently with the activation of microglia and astrocytes. Assessing activation at this early stage could be used as a diagnostic tool. Some success with miR-155 targeting has been reported in the SOD1 mouse, a model for amyotrophic lateral sclerosis (ALS). Targeting of miR-155 contributed to the reduction of inflammation and a corresponding reduction in the severity of the phenotype (67). This provides encouraging evidence that miR-155 may be a key player in many different neurodegenerative diseases beyond ALS and AD. miRNAs will prove to be fertile ground for the study of neurodegenerative diseases far into the future.

### Long Non-Coding RNA

Broadly defined, lncRNAs are RNA transcripts, which are more than 200 nt long and do not code for proteins, and are usually found near protein-coding regions. An excellent review by Kung and colleagues categorizes lncRNAs based on their genomic context, although they are quick to note that these categorizations do not provide much information on function or history of the lncRNA (68). It is these diverse functions that make the subject of lncRNA so interesting to researchers, with recent reviews covering the role of lncRNA in cancer (69), aging in the brain (70), and autoimmune disease (71). This section of the review summarizes the role of lncRNAs as they pertain to monocytes in steady state and inflammation.

While a host of factors control the complex process of cell differentiation, some researchers have identified specific lncRNAs that are essential to the differentiation of monocytes into their functional descendants. For example, in order for DC to differentiate from monocytes, the lncRNA lnc-DC needs to activate signal transducer and activator of transcription 3 (STAT3), a transcription factor (72). Lentivirus-mediated RNA interference to knockdown lnc-DC caused a downregulation of many genes related to DC function and caused the monocyte marker CD14 to be upregulated in these DC. In the cell line U937, a model human cell line in which monocytes can be differentiated into macrophages with the application of phorbol myristate acetate (PMA), cells with the lncRNA P50-Associated COX-2 Extragenic RNA (PACER) knocked down do not differentiate into macrophages upon treatment (73). PMA-treated knocked down cells also had an attenuated response to LPS compared to PMA-treated controls. PACER was shown to control the cyclooxygenase 2 (COX-2) gene, whose overexpression is implicated in a variety of cancers. Zhang and colleagues found that the lncRNA HOX antisense intergenic RNA myeloid 1 (HOTAIRM1) affects the transcription of HOXA genes, which impacts the expression of CD11b and CD18, genes that are hallmarks of granulocyte maturation (74). lncRNA may be involved in the widely studied phenomenon of changes in monocyte genomic transcription in response to inflammation. Illott and colleagues (75) found that LPS stimulation of human monocytes caused 221 lncRNAs to be differently expressed. Knockdown of enhancer regions associated with some of these transcripts caused attenuation of the LPS-induced release of proinflammatory IL1b and CXCL8. It has also been shown that THRIL (TNFα and hnRNPL related immunoregulatory LincRNA) regulates the expression of TNFalpha in THP1 macrophages (76). Reddy and colleagues found that in diabetic mice, the lncRNA E330013P06 (E33) is upregulated in macrophages. In an *in vitro* study, macrophages overexpressing E33 and exposed to LPS expressed more IL-6, TNF, CD36, and CCL2 than controls (77) Thus, this lncRNA is part of a positive feedback loop of increasing inflammation in a disease model. This illustrates the potential for lncRNA as a target for therapies.

With next generation sequencing techniques creating a huge volume of data on lncRNA, it is becoming increasingly possible to filter signal from noise. Future researchers will thus be able to design in depth studies on function of these unique RNAs.

### RNA-Binding Proteins

Monocytic cells contain a diverse repertoire of RBPs that govern the fate of transcripts by mediating mRNA processing (55). The RBPs control mRNA stability and translation in response to various stimuli (e.g., developmental signaling, stress, and immune challenges) through selective interaction with 3′ untranslated regions (UTR) of their target mRNA (55, 56). In monocytic cells, these RBP–RNA complexes (ribonucleoprotein complexes; RNP complexes) provide more rapid and flexible gene expression for inflammation through post-transcriptional regulation.

RNA-binding protein complexes are composed of transcripts that contain adenine and uridine-rich elements (AREs) and the proteins that bind the AREs (ARE–RBPs) (78, 79). AREs recruit many ARE–RBPs that modulate the stability of target mRNAs, and their translation. Each ARE–RBP has a distinct regulatory function on mRNA stability and translation in cells of monocytic lineage. For example, tristetraprolin (TTP) destabilizes mRNA of TNF-α and inhibits TNF-α production in LPS-stimulated macrophages (80, 81). In contrast, human antigen R (HuR) increases TNF-α mRNA stability and reduces its translation in LPS-stimulated macrophages by interfering with the functions of T-cell-restricted intracellular antigen 1 (TIA-1) and TTP (82). Moreover, AU-binding factor 1 (AUF-1) deficiency enhanced macrophage recruitment to the sites of inflammation without direct degradation of target mRNA (56, 83). These results imply a distinct regulatory role of the ARE–RBP complex in the coordination of the stability and translation of mRNA in monocytic cells.

In addition to ARE–RBPs complexes, the interferon (IFN)-γ-activated inhibitor of translation element (GAIT) suggested post-transcriptional regulation that functions to limit or resolve inflammation (55, 84). In monocytic cells, IFN-γ can induce either pro- or anti-inflammatory responses depending on the context. Generally, IFN-γ stimulation induces formation of the heterotetrameric GAIT complex in 3′ UTR region, which consists of glutamyl-prolyl tRNA synthetase (EPRS), NS1-associated protein 1 (NSAP1), ribosomal protein L13a, and glyceraldehyde-3-phosphate dehydrogenase (GAPDH). Recent genome-wide microarray analysis of IFN-γ-activated monocytic cells identified a family of mRNAs encoding multiple chemokine ligands and receptors, as candidate GAIT pathway targets (85). Indeed, this study revealed the GAIT complex is able to silencing multiple transcripts encoding inflammatory molecules (CCL22, CCR3, CCR4, and CCR6, and apolipoprotein L2) in L13a-dependent manner (85).

The binding activity of RBPs on their target transcript can be modulated by cofactors as translational activators or repressors. Although literature identifying cofactors in RBPs mediating post-transcriptional regulation is very poor in monocytic cells, Yu and colleague showed that steroid receptor co-activator 3 (SRC3) enhanced TIA-1’s repressive effect on TNF-α mRNA translation in LPS-stimulated macrophages (86). Moreover, macrophages obtained from SRC3-deficient mice produced significantly more proinflammatory cytokines, such as TNF-α, IL-6, and IL-1β, than wild-type controls. In light of these observations, SRC-3 can repress cytokine production by influencing the binding of TIA-1/TIAR to ARE-containing transcripts at the mRNA translational level in monocytic cells.

### RNA Editing

RNA editing is one of main categories of RNA modifications that code dynamic regulatory information on mRNA and non-coding RNA on 5′ and 3′ UTR. Editing is catalyzed by two classes of deaminases: those that convert adenosine to inosine (adenosine deaminase acting on RNA, ADARs) and those that convert cytosine to uracil (apolipoprotein B mRNA editing enzyme, catalytic polypeptide, APOBECs) (58, 59). A-to-I RNA editing can affect not only the coding portions of pre-mRNAs but also the non-coding regions: the 5′- UTRs, the 3′-UTRs and introns. Similar with C to U editing by ADARs, APOBECs are also known to modify coding portions and 3′ UTR of target transcript sequence. Especially, modification on 3′ UTR by ADARs and APOBEC1 may block miRNA binding, or introduce new miRNA seed target sequences, or shift existing targets to sequences that recruit different miRNA (58, 59).

Although little is known about the RNA editing by ADARs and Apobec1 in monocytes, it has been hypothesized that Apobec-1 mediating RNA editing is associated with post-transcriptional regulation by RBPs in monocytes. Gene array analysis has demonstrated that TIA-1-induced translational silencing concomitantly promotes the decay of Apobec1 mRNA in peritoneal macrophages (87). In addition to transcriptional interaction between TIA-1 and Apobec1, Apobec1 was present in TIA-1-positive RNA granules *in vitro*, indicating that Apobec1 may participate in the mRNA metabolism alterations mediated by RNA granules (88).

## Diseases Associated with Epigenetic Modulation of Monocytic Cells

The mature nervous system is a dynamic and plastic anatomic entity comprising multiple cell types that communicate through both “hardwiring” and the release of signaling molecules stimulated by local, distant, and external environmental cues. These signaling pathways in turn orchestrate the many complex functions needed to pilot the body throughout life and include behavior, learning and memory, reproduction, and immune-mediated protection. While such complex functions are developmentally established by an anatomic structural blueprint, it is now clear that fine-tuning of these communication processes is required at every level to adapt to the variability and diversity of cellular or external environmental cues.

Epigenetic mechanisms play a major role in generating cell type diversity in the CNS by influencing the architecture of chromosomes as well as the activation and repression of genetic information during critical developmental stages. Genetic anomalies affecting various components of epigenetic machinery have been shown to induce changes in neural cell identity as well as in cognitive and behavioral phenotypes and may underlie the pathophysiological diversities observed in the spectrum of neurological diseases. The epigenetic mechanisms as touched upon in this review provide a major way for this fine-tuning to occur in cells of the mononuclear phagocyte system. While this field is still in its infancy, it has evolved enough to require its inclusion in our thinking about the etiologies of CNS diseases with regards to epigenetic “fine tuning” mechanisms and the cumulative, long range consequences they may have when they become “dysfunctional.”

When you want to understand a mechanism, study a disease – Robert Good (circa 1985)

There are several reports in the literature related to cells of the monocytic lineage that have opened the door to new understanding of epigenetic mechanisms underlying some disease states of the CNS. We now recognize two new factors that lead us to reconsider the phenotypic and functional heterogeneity of cells previously referred to as “microglia”: (i) leukocytes, monocytes, in particular, readily have access to the CNS and can carry out immune functions of surveillance and tolerance in the steady state (4) and (ii) the origin and immune role of the “microglia” has become less clear in the steady state and following neuronal damage (89–92).

In addition to the better studied epigenetic processes (DNA methylation, histone modifications), which are postulated to dynamically transduce environmental inputs into lasting physiological and behavioral changes, we propose that the epitranscriptomic process of RNA editing in response to stimuli as diverse as stress, infection, or hormonal stimulation may be the bases of cellular and functional diversity seen within the cells of monocytic lineage in the CNS and beyond.

Both ADAR-mediated and APOBEC-mediated RNA editing events have been cataloged from a variety of tissues, including cells of the monocytic lineage (93). Within the populations of cells of the monocytic lineage, robust editing has been detected from RNA-seq data in hundreds of distinct transcripts, but editing rates vary widely (from under 1% to almost 100%). This range of editing rates could either result from approximately equal rates of editing within each individual cell in the population, or could instead correspond to an average of distinct, variable editing signatures across individual cells in the population. Our recent data explicitly support the hypothesis that RNA editing generates diverse cellular populations with distinct signatures Harjanto et al., in revision. Thus, editing-mediated RNA-level sequence diversity may contribute to the functional heterogeneity apparent in immune cell populations at steady state.

Direct reports of epigenetic events associated with the brain function or pathology have been carried out for the most part on selected human postmortem CNS tissue, which lend to experimental difficulties in the execution and interpretations of the data. The clearest reports of epigenetic RNA editing associated with brain function have been limited to selected regions of the CNS rather than on any specific cell type. Gaisler-Solomon et al. have shown A-to-I editing, which is catalyzed by ADAR2, to occur at the Q/R site of the α-amino-3-hydroxy-5-methyl-4-isoxazolepropionic acid (AMPA) glutamate receptor subunit GluA2 (94). Changes leading to the reduction of editing at this site led to abnormal calcium fluxes and cell death. Examination of hippocampal tissue derived from AD patients showed marked decrease in editing events at the GluA2 site compared to control tissue. This effect was also seen in tissue derived from subjects carrying the apolipoprotein E4 allele regardless of their clinical diagnosis. In addition, ADAR2 mRNA was reported to be decreased in the AD caudate tissue, which collectively suggested to these authors that changes in these editing events correlates with neurodegenerative disease. These data are in line with previous reports (95–98), suggesting that ADAR2-dependent GluR2/GluA2 editing sites in neurons may be vulnerable to pathological states. Although in these studies neurons were shown to be the site of editing of AMPA receptor subunit, monocytic cells isolated from glioblastomas have also known to express and upregulate the expression of GRIA2 (GluA2 or AMPA receptor 2) (99). While not investigated, it is entirely possible that these monocytic cells may also edit the GluA2 subunit via this pathway, which may be a contributing factor in the neurobiology of this CNS disease.

Baysal et al. (100) have shown that C-to-U RNA editing of (C136U, R46X) in monocytes by APOBEC1 inactivates a small fraction of succinate dehydrogenase (SDH; mitochondrial complex II) subunit B (SDHB) mRNAs in normal steady-state peripheral blood mononuclear cells. Mutations in SDH, a heterotetrameric tumor suppressor complex, cause paraganglioma tumors that are associated with activation of hypoxia inducible pathways. This study found that C–U editing was down regulated in mRNA of this peptide during hypoxia events, specifically suggesting C–U editing by APOBEC1 helps monocytes to adapt to hypoxic conditions and more broadly suggests that C-to-U recoding of the RNA of certain genes is dynamically induced by physiologically relevant environmental factors. Most recently, these data have been extended to human monocytes. Sharma et al. (93) have demonstrated that C–U RNA editing by APOBEC3A (cytidine deaminase) of innate restriction factors occurs in macrophages during M1 polarization as well as in monocytes in response to hypoxia and interferons.

Studies from our laboratories show that RNA editing by APOBEC1 in bone marrow-derived mouse macrophages lead to the generation of populations that are heterogeneous and functionally diverse, enabling rapid population adaption in different environmental settings. We have further demonstrated *in vivo* using the BV2 microglia cell line and *ex vivo* using wildtype and APOBEC1 knockout mouse that APOBEC 1 catalyzed editing occurs in brain cells of monocytic lineage. Additionally, we have shown that APOBEC1 in brain-derived monocytes is targeted by a rhabdovirus to maintain an intracellular, steady-state environment in order to utilize its cellular machinery and facilitate viral replication Chung et al., under review.

It has been shown that editing rates tend to increase with increased expression of the cognate enzyme (101). Expression of both editing enzymes increases under inflammatory conditions (101, 102) and in the cases described thus far (in the context of cancer) increased editing correlates with increased progression and poor prognosis (101, 102). In light of these reports, we can hypothesize that editing enzymes may play critical role in the etiology of CNS diseases, many of which are associated with inflammatory conditions. We believe that a better understanding of the functional outcomes of epigenetic modifications, such as RNA editing, and of diseases specifically associated with hyper- or hypo-editing will be of substantial interest in many neuropathologies of unknown etiologies which show significant “weak or missing heritability” at the genetic level, but which clearly have a strong genetic component.

## Author Contributions

All authors contributed equally to the writing of the manuscript. FP and KB edited the manuscript.

## Conflict of Interest Statement

The authors declare that the research was conducted in the absence of any commercial or financial relationships that could be construed as a potential conflict of interest.
